# Solubility of hesperidin drug in aqueous biodegradable acidic choline chloride-based deep eutectic solvents

**DOI:** 10.1038/s41598-023-38120-x

**Published:** 2023-07-12

**Authors:** Hemayat Shekaari, Mohammed Taghi Zafarani-Moattar, Masumeh Mokhtarpour, Saeid Faraji

**Affiliations:** Department of Physical Chemistry, University of Tabriz, Tabriz, Iran

**Keywords:** Chemical engineering, Physical chemistry

## Abstract

Important efforts have been made over the past years to improve the drug acts, which leads to the discovery of novel drug preparations and delivery systems. The selection of suitable green solvents for novel drug discovery and drug delivery depends on a molecular-level understanding of the interaction between drug molecules and the solvents. Deep eutectic solvents (DESs) are already used in sustainable extraction methods of natural products for their very high solvent power, high chemical and thermal stability, non-toxicity, and non-flammable. The thermodynamic investigation provides deep and complete knowledge of interactions and the choice of appropriate and suitable production compounds in pharmaceutical fields. Particularly, the analysis of drugs+DESs in aqueous media is a central issue in many types of research. This research is aimed to determine hesperidin (HES) solubility in water and DES solvents [choline chloride/citric acid (ChCl/CA), choline chloride/oxalic acid (ChCl/OA), choline chloride/malonic acid (ChCl/MA), and choline chloride/lactic acid (ChCl/LA)] at temperature range (298.15–313.15 K). Furthermore, the measured solubility data of HES in studied aqueous DESs solutions was fitted by models of Van’t Hoff–Jouyban–Acree and Modified Apelblat–Jouyban–Acree. Finally, the Hansen solubility parameters as thermodynamic aspect for analyzing the dissolution processes for the four investigated aqueous DESs solutions were estimated.

## Introduction

**Figure 1:**
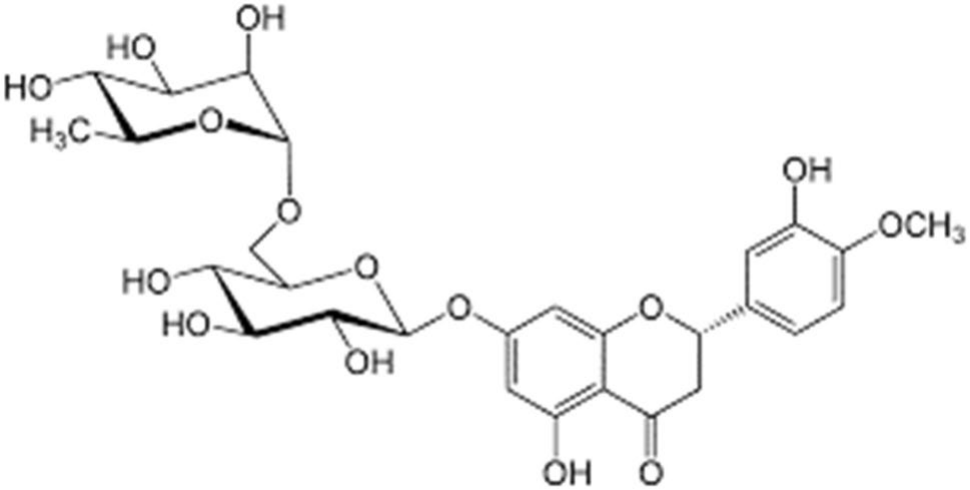
Molecular structure of hesperidin.

Hesperidin (HES) is a flavanone glycoside (molecular structure shown in Fig. 1) found in citrus fruits and citrus fruit-derived products that is commonly found in diet^1–3^. It has been extensively used in antioxidant, anticancer, anti-inflammatory, and antimicrobial activities^4^. However, its low aqueous solubility is a crucial drawback in formulation development^5^ and it has low bioavailability because of poor absorption in the small intestine^6,7^.

According to the Biopharmaceutical Classification System (BCS), drugs are classified into four major groups based on solubility and permeability^8^. This classification's most important group is appropriate permeability and low water solubility. As a result, improving drug solubility is becoming more important the drug’s solubility in the pharmaceutical industry. Consequently, enhancement of the drugs solubility is more important in pharmaceutical industries. The HES is one of these categories which have low solubility in aqueous systems and proper permeability. The co-solvency method has been demonstrated to be a practical and effective method for increasing a drug's solubility in water. The co-solvents are separated into several significant categories, including organic solvents, ionic liquids (ILs), and deep eutectic solvents (DESs). The organic solvents applied in numerous systems are usually volatile, toxic, and flammable. The ILs are a class of salts that are expected to be used as an innovative source of solvents and for several other applications. However, these solvents are expensive and hard to prepare^9,10^. In last decade, DESs have received great attention in many fields of sciences because of their unique properties including biodegradability, biocompatibility, low-cost and easy preparation process^10–12^. These solvents commonly refer to the transparent homogeneous liquids made by taking two or more compounds as a hydrogen bond acceptor (HBA) and a hydrogen bond donor (HBD) through strong intermolecular interactions^13^. On the other hand, the acidic DESs including natural acidic compounds have received great attention owing to their distinct superiorities. Their properties (such as acidity, density, viscosity, etc.) changes depending on both the nature and ratio of the counterparts, which can be tailored according to the specific applications. Recently, the applications of DESs have been presented in several works^14–17^.

Various methods (including micro-particles, complexation, and nanocrystals^5,18,19^) have been used to enhance the solubility and bioavailability of HES. In the earlier works, only the solubilities of HES in some organic solvents including ethanol, water, isopropanol, 1-butanol, propylene glycol, and PEG-400 are available^20^. From a practical standpoint, drug solubility in co-solvent solutions is critical in material purification, dosage form design, and understanding the mechanism governing the chemical and physical stability of pharmaceutical solutions. As a result, determining drug solubility in mixed solvents is critical^21^.

This work is aimed to investigate the solubility of the HES in the presence of some acidic DESs based on choline chloride (ChCl) as HBA and citric acid (CA), oxalic acid (OA), malonic acid (MA) and lactic acid (LA) as HBDs in various mass fraction of DESs at atmospheric pressure and at temperature range *T* = (298.15–313.15 K). Then the obtained solubility data have been correlated by using models such as Van’t Hoff–Jouyban–Acree and Modified Apelblat–Jouyban–Acree models. Moreover, the proper choice of solvent can be done by the Hansen solubility parameters (HSP) and the Hildebrand solubility parameters, which are empirical methods. Both the total solubility parameter^22^ and its constituent partial solubility parameters (HSP)^23,24^ are widely used to study the effect of solvent on solute solubility. In the next step, the Hansen parameters were used to select the best solvent for the studied drug, and the obtained results were compared with the experimental results.

## Experimental

### Materials

Hesperidin, choline chloride, citric acid, oxalic acid, malonic acid, lactic acid, and sodium hydroxide were employed. The detailed information about the materials is listed in Table 1.

**Table 1:** Some information; chemical name, provenance, CAS No., molar mass and mass fraction (purity) of the used materials.

Chemical name	Provenance	Molar mass (g mol^−1^)	CAS No	Mass fraction (purity)
Hesperidin	Sigma-aldrich	610.19	520-26-3	≥ 0.85
Choline chloride	Merck	139.62	67-48-1	> 0.99
Oxalic acid	Merck	90.03	144-62-7	> 0.99
Malonic acid	Merck	104.06	141-82-2	> 0.99
Lactic acid	Merck	90.08	50-21-5	> 0.99
Citric acid	Merck	192.12	77-92-9	> 0.99

The suppliers were provided the purities of the used components.

### Apparatus and procedure

#### Preparation of ChCl-based DESs

An analytical balance with a precision of 10^–4^ g was used to prepare deep eutectic solvents (DESs) (AW 220, GR220, Shimadzu, Japan). Acidic DESs based on choline chloride as HBA and citric acid, oxalic acid, malonic acid, and lactic acid as HBDs were made by combining specific molar ratios of HBA: HBD^25–27^. The mixtures were stirred at 363.0 K (temperature higher than their melting points) until they were colorless, homogeneous, and clear. The solvents were then dried at room temperature using a vacuum pump. The water content of prepared DESs was determined using the 751GPD Titrino-Metrohm Karl-Fischer titration (method TitroLine KF). Table 2 lists the thermophysical properties of the DESs.

**Table 2:** Common properties of DESs used in this work at 298.15 K and 0.0871 MPa

Name	DES abbreviation	Salt—HBD (Molar ratio)	Water content	Molar mass (g mol^−1^)
Choline chloride/oxalic acid	ChCl/OA	1:1	0.09%	114.826
Choline chloride/malonic acid	ChCl/MA	1:1	0.09%	114.826
Choline chloride/lactic acid	ChCl/LA	1:2	0.07%	106.594
Choline chloride/citric acid	ChCl/CA	1:1	0.05%	165.871

Standard uncertainty for pressure u(*P*) = 0.0001 MPa.

#### Determination of HES solubility using HPLC

**Figure 2:**
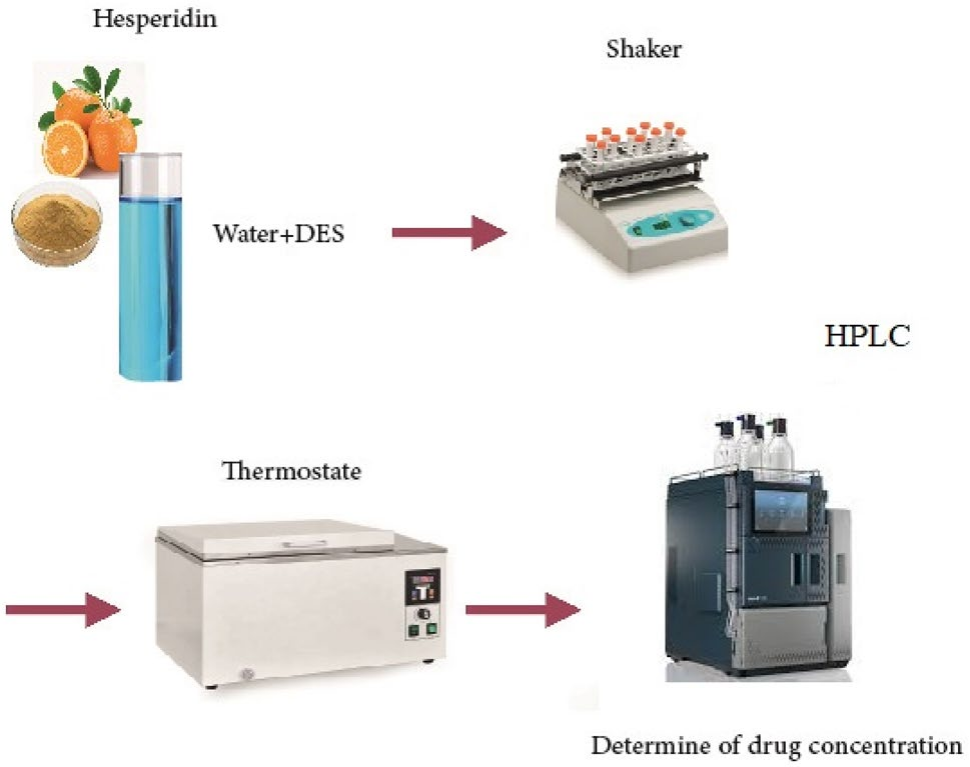
Schematic of the measuring process of the sample’s solubility.

Using the saturation shake-flask method, the solubility of HES in the chosen solvents (DES + water) was determined (Fig. 2)^28–30^. For this purpose, the experimental steps are as follows:

1. Take 2 g of solvents mixtures and add it to glass tubes.
2. Turn on the constant temperature water bath and magnetic stirring to reach the required temperature.
  At this temperature, the HES sample was added several times with stirring until a precipitate appeared, which was no longer dissolved after 3 h of stirring. At this point, it can be considered that the solid–liquid equilibrium is reached, and it is allowed to stand for 48 h.
3. Then the supernatant solutions were filter through a 0.45 μm membrane (Durapore® membrane filters, type HV, 0.45 μm, Millipore, MA).
4. Drug uptake in diluted samples (using NaOH solutions with 20% mass percent) were analyzed by reported high performance liquid chromatography (HPLC) at a detection wavelength of 346 nm after suitable dilution with mobile phase.
  The calculation equations for the solubility data of HES (*x*_1_) in solvents are as follows^31,32^:
  1 $$x_{1} = \frac{{m_{1} /M_{1} }}{{m_{1} /M_{1} + m_{2} /M_{2} + m_{3} /M_{3} }}$$
  herein *m*_i_ and *M*_i_ are the mass (g) and molar mass (g mol^−1^) of HES (1), water (2), DES (3), respectively.

#### Prediction of HES solubility using Hansen solubility parameters (HSP)

**Figure 3:**
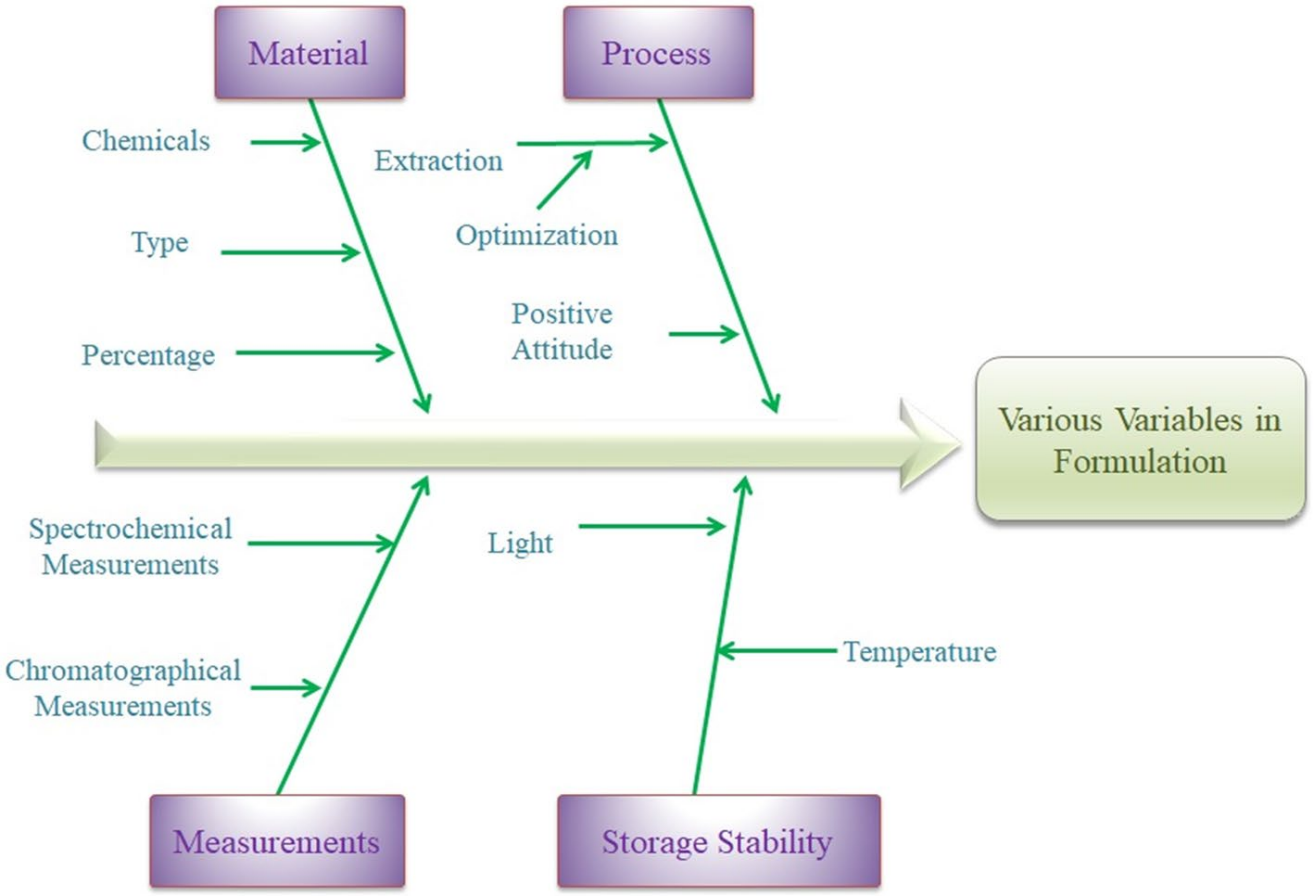
Fish-Bone diagram for the variables in formulation of drugs.

In any solvation and dissolving process, the choice of an appropriate solvent is a crucial issue. The selection of appropriate solvents for drug solubilization is based on each drug solubility in the respective solvent or mixture of solvents. Some parameters can affect the process of the drugs formulation (Fig. 3). The solute–solvent interactions in systems can be predicted using a various technique, but the Hansen solubility parameters (HSP) offer a measurable way to estimate how soluble one material is in another. Hildebrand was the first to introduce solubility parameters, confirming the statement that “similar solves similar”^33^. Hansen^34^ completes this empirical parameter, which is used as the Hildebrand-Hansen solubility parameter. The following relationship can be used to calculate the solubility parameters:

2 $$\delta^{2} = \frac{{E_{coh} }}{{V_{m} }} = \frac{{\Delta H_{vap} - RT}}{{V_{m} }}$$

where *E*_coh_, *V*_m_ and Δ*H*_vap_ are the intermolecular forces (adhesion energy), the molar volume and the evaporation enthalpy, respectively. In addition, *R* and *T* represent the general constant of the gases and the temperature (K).

The interactions between the solute and the solvent in the investigated systems are described by Hansen solubility parameters, which are more complicated three-dimensional solubility parameters. The sum of the energies required to overcome scattering forces (*δ*_d_), adjacent intermolecular forces (bipolar interactions) (*δ*_p_), and molecule-to-molecule hydrogen bond failure (*δ*_h_) is calculated as the adhesion energy density:

3 $$\delta_{t}^{2} = \delta_{d}^{2} + \delta_{p}^{2} + \delta_{h}^{2}$$

The mutual solubility between solute *i* and solvent *j* is calculated from the following equation:

4 $$\Delta \delta_{ij} = \sqrt {4\left( {\delta_{d}^{i} + \delta_{d}^{j} } \right)^{2} + \left( {\delta_{p}^{i} + \delta_{p}^{j} } \right)^{2} + \left( {\delta_{h}^{i} + \delta_{h}^{j} } \right)^{2} }$$

The methods based on contribution of different functional groups are employed to estimate *δ*_h_, *δ*_p_, and *δ*_d_. Thus, *δ*_d_ is obtained from the following equation:

5 $$\delta_{d} = \frac{{\sum {F_{d} } }}{{V_{m} }}$$

where *F*_d_ is the constant dispersion component of molar adsorption. The interactions of polar groups are also expressed with the help of the following relation:

6 $$\delta_{p} = \frac{{\sum {F_{p}^{2} } }}{{V_{m} }}$$

here, *F*_p_ is the constant polar component of molar adsorption. *δ*_h_ can also be calculated from the following equation:

7 $$\delta_{h} = \frac{{\sum {E_{h} } }}{{V_{m} }}$$

where *E*_h_ is the hydrogen bond adhesion energy per structural group. Using the literature^35^, we can calculate the solubility parameters for different materials.

For the employed DESs, which are collected in Table 3, the parameters *δ*_d_, *δ*_p_ and *δ*_h_ were obtained from the literature, and some were obtained using the Van Krevelen-Hoftyzer approach^36^.

**Table 3:** Experimental mole fraction solubility ($x_{1}^{exp}$) and calculated solubility ($x_{1}^{cal}$) of HES in the aqueous DES solutions at various temperatures (*T*) and weight fractions of DES (*w*_3_) at *P* = 871 hPa.

*T*/K	$x_{1}^{exp}$	$x_{1}^{cal}$	
		Van’t Hoff–Jouyban–Acree	Modified Apelblat–Jouyban–Acree
HES + water + ChCl/OA			
* w*_3_ = 0.00	$10^{7} x_{1}^{exp}$		
298.15	1.4201	–	–
303.15	1.5299	–	–
308.15	1.5796	–	–
313.15	1.6409	–	–
*w*_3_ = 0.02	$10^{6} x_{1}^{exp}$	$10^{6} x_{1}^{cal}$	$10^{6} x_{1}^{cal}$
298.15	5.5812	5.5832	5.5837
303.15	5.9804	5.9810	5.9754
308.15	6.2331	6.2388	6.2349
313.15	6.4801	6.4842	6.4801
* w*_3_ = 0.05	$10^{6} x_{1}^{exp}$	$10^{6} x_{1}^{cal}$	$10^{6} x_{1}^{cal}$
298.15	5.8969	5.8689	5.8675
303.15	6.0229	6.0416	6.0334
308.15	6.4602	6.4281	6.4239
313.15	6.7504	6.7416	6.7371
* w*_3_ = 0.07	$10^{6} x_{1}^{exp}$	$10^{6} x_{1}^{cal}$	$10^{6} x_{1}^{cal}$
298.15	6.1209	6.1519	6.1494
303.15	6.3511	6.3353	6.3255
308.15	6.5601	6.6118	6.6074
313.15	6.9216	6.9376	6.9329
* w*_3_ = 0.10	$10^{6} x_{1}^{exp}$	$10^{6} x_{1}^{cal}$	$10^{6} x_{1}^{cal}$
298.15	6.5411	6.5274	6.5239
303.15	6.7808	6.7996	6.7879
308.15	6.8811	6.8614	6.8563
313.15	7.1801	7.1757	7.1708
* w*_3_ = 0.15	$10^{6} x_{1}^{exp}$	$10^{6} x_{1}^{cal}$	$10^{6} x_{1}^{cal}$
298.15	6.8902	6.8912	6.8889
303.15	6.9811	6.9899	6.9767
308.15	7.2112	7.2156	7.2107
313.15	7.4604	7.4631	7.4579
HES + water + ChCl/MA			
* w*_3_ = 0.02	$10^{6} x_{1}^{exp}$	$10^{6} x_{1}^{cal}$	$10^{6} x_{1}^{cal}$
298.15	4.4499	4.4497	4.4496
303.15	4.8511	4.8504	4.8499
308.15	4.9909	4.9912	4.9915
313.15	5.2102	5.2000	5.2025
* w*_3_ = 0.05	$10^{6} x_{1}^{exp}$	$10^{6} x_{1}^{cal}$	$10^{6} x_{1}^{cal}$
298.15	4.6804	4.6634	4.6631
303.15	4.9511	4.9507	4.9492
308.15	5.3402	5.3307	5.3309
313.15	5.5498	5.5994	5.6027
* w*_3_ = 0.07	$10^{6} x_{1}^{exp}$	$10^{6} x_{1}^{cal}$	$10^{6} x_{1}^{cal}$
298.15	4.8914	4.9049	4.9045
303.15	5.1212	5.1234	5.1213
308.15	5.4708	5.4825	5.4828
313.15	5.8909	5.8150	5.8188
* w*_3_ = 0.10	$10^{6} x_{1}^{exp}$	$10^{6} x_{1}^{cal}$	$10^{6} x_{1}^{cal}$
298.15	5.2500	5.2368	5.2363
303.15	5.3801	5.3825	5.3796
308.15	5.6218	5.6142	5.6145
313.15	6.0106	6.0405	6.0449
* w*_3_ = 0.15	$10^{6} x_{1}^{exp}$	$10^{6} x_{1}^{cal}$	$10^{6} x_{1}^{cal}$
298.15	5.5401	5.5357	5.5355
303.15	5.6513	5.6541	5.6499
308.15	5.8304	5.8305	5.8309
313.15	6.4500	6.4444	6.4499
HES + water + ChCl/LA			
* w*_3_ = 0.02	$10^{6} x_{1}^{exp}$	$10^{6} x_{1}^{cal}$	$10^{6} x_{1}^{cal}$
298.15	4.2111	4.2089	4.2047
303.15	4.3597	4.3518	4.3521
308.15	4.7508	4.7414	4.7404
313.15	4.9315	4.9268	4.9272
* w*_3_ = 0.05	$10^{6} x_{1}^{exp}$	$10^{6} x_{1}^{cal}$	$10^{6} x_{1}^{cal}$
298.15	4.4217	4.4406	4.4359
303.15	4.5708	4.6132	4.6138
308.15	4.8616	4.9032	4.9003
313.15	5.1211	5.1395	5.1402
*w*_3_ = 0.07	$10^{6} x_{1}^{exp}$	$10^{6} x_{1}^{cal}$	$10^{6} x_{1}^{cal}$
298.15	4.6400	4.6165	4.6114
303.15	4.8923	4.8242	4.8250
308.15	5.1731	5.1087	5.1048
313.15	5.3614	5.3328	5.3337
* w*_3_ = 0.10	$10^{6} x_{1}^{exp}$	$10^{6} x_{1}^{cal}$	$10^{6} x_{1}^{cal}$
298.15	4.8111	4.8244	4.8190
303.15	5.0504	5.0767	5.0778
308.15	5.3718	5.3992	5.3940
313.15	5.5807	5.5926	5.5938
* w*_3_ = 0.15	$10^{6} x_{1}^{exp}$	$10^{6} x_{1}^{cal}$	$10^{6} x_{1}^{cal}$
298.15	5.0101	5.0117	5.0057
303.15	5.2600	5.2543	5.2561
308.15	5.6802	5.6790	5.6721
313.15	5.9811	5.9797	5.9809
HES + water + ChCl/CA			
* w*_3_ = 0.02	$10^{5} x_{1}^{exp}$	$10^{5} x_{1}^{cal}$	$10^{5} x_{1}^{cal}$
298.15	1.5513	1.5488	1.5499
303.15	1.8301	1.8297	1.8314
308.15	2.2204	2.2084	2.2078
313.15	2.5612	2.5547	2.5546
* w*_3_ = 0.05	$10^{5} x_{1}^{exp}$	$10^{5} x_{1}^{cal}$	$10^{5} x_{1}^{cal}$
298.15	1.8411	1.8419	1.8436
303.15	1.9821	1.9767	1.9794
308.15	2.3601	2.4246	2.4229
313.15	2.8908	2.9151	2.9146
* w*_3_ = 0.07	$10^{5} x_{1}^{exp}$	$10^{5} x_{1}^{cal}$	$10^{5} x_{1}^{cal}$
298.15	1.9507	1.9445	1.9466
303.15	2.1309	2.1314	2.1348
308.15	2.7811	2.6793	2.6768
313.15	3.2103	3.1697	3.1689
* w*_3_ = 0.10	$10^{5} x_{1}^{exp}$	$10^{5} x_{1}^{cal}$	$10^{5} x_{1}^{cal}$
298.15	2.0104	2.0106	2.0131
303.15	2.3416	2.3365	2.3409
308.15	2.9805	3.0285	3.0250
313.15	3.4501	3.4674	3.4662
* w*_3_ = 0.15	$10^{5} x_{1}^{exp}$	$10^{5} x_{1}^{cal}$	$10^{5} x_{1}^{cal}$
298.15	2.2400	2.2383	2.2419
303.15	2.4511	2.4476	2.4532
308.15	3.1400	3.1365	3.1319
313.15	3.7808	3.7773	3.7756

### Thermodynamic analysis

#### Solubility modeling

Solubility is the most crucial element in the development of pharmacological drugs. Drug solubility cannot always be assessed across the whole range of solvent temperatures or concentrations. Additionally, some theoretical models can be used to fit the solubility of pharmaceutical compounds in various systems in a given region and then forecast the solubility of the compounds in other concentration and temperature ranges, saving time and money during the experimental procedure.

#### Van’t Hoff–Jouyban–Acree model

The Van’t Hoff equation is another model that represents the dependence of the natural logarithm of mole fraction solubility on absolute temperature.

8 $$\ln x_{T} = A + \frac{B}{T}$$

Using the Eq. (8), the Van’t Hoff–Jouyban—Acree model can be derived^37^ and expressed as Eq. (9).

9 $$\log X_{1,T} = w_{2} \left( {A_{2} + \frac{{B_{2} }}{T}} \right) + w_{3} \left( {A_{3} + \frac{{B_{3} }}{T}} \right) + \frac{{w_{2} w_{3} }}{T}\sum\limits_{i = 0}^{2} {J_{i} (w_{2} - w_{3} )^{i} }$$

*A*_2_, *B*_2_, *A*_3_, *B*_3_ and *J*_i_ are the model parameters.

#### Modified Apelblat–Jouyban–Acree model

The Modified Apelblat model is a semi-empirical model. The relationship between temperature and solubility can be studied by using this model^38,39^:

10 $$\ln x_{T} = A + \frac{B}{T} + C\ln T$$

where *A*, *B*, and *C* are equation parameters; and also, *x*_T_ is the mole fraction solubility of HES in solvent mixtures at temperature *T* in Kelvin. The Modified Apelblat–Jouyban–Acree model is as follow^40^:

11 $$\log X_{1,T} = w_{2} \left( {A_{2} + \frac{{B_{2} }}{T} + C_{2} \ln T} \right) + w_{3} \left( {A_{3} + \frac{{B_{3} }}{T} + C_{3} \ln T} \right) + \frac{{w_{2} w_{3} }}{T}\sum\limits_{i = 0}^{2} {J_{i} (w_{2} - w_{3} )^{i} }$$

The average relative deviation percent (*ARD%*), which is produced for the applied models using the formula given below, is used to describe the discrepancy between the experimental and calculated solubility results:

12 $$ARD = 100\left( {\frac{{\sum\nolimits_{i = 1}^{N} {\frac{{\left| {x_{i}^{\exp } - x_{i}^{cal} } \right|}}{{\left| {x_{i}^{\exp } } \right|}}} }}{N}} \right)$$

where $x_{i}^{\exp }$, $x_{i}^{cal}$ and *N* are experimental and calculate solubility data and number of experimental points, respectively.

## Results and discussion

### Solubility data

**Figure 4:**
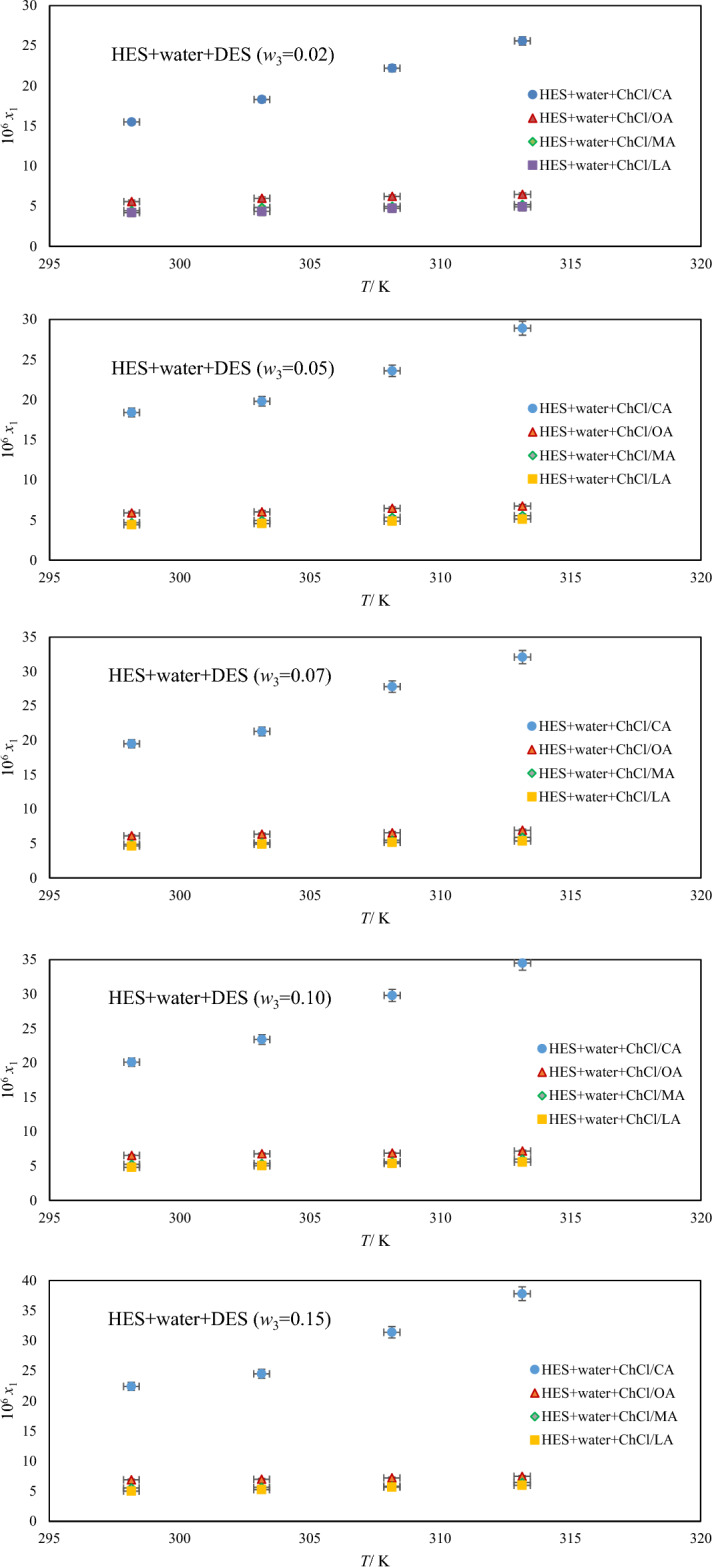
The solubility mole fraction of HES versus temperatures in aqueous DES solutions.

The solubility of HES in four selected aqueous quasi-binary solvents (water+DESs) was determined experimentally in a series of weight fractions of DES (0.00, 0.02, 0.05, 0.07, 0.10, 0.15) at temperature intervals of 5 K ranging from 298.15 to 313.15 K. The solubility results are collected in Table 3 and graphically is shown in Fig. 4 for *T* = 298.15 K. As shown in Fig. 4, increasing the weight fraction (*w*_3_) of DES improves HES solubility, whereas at a constant weight fraction composition (*w*_3_), HES solubility increases with increasing temperature. According to Table 3, DESs as green co-solvents appear to improve HES solubility more than pure water. These findings indicate that the used DESs increase the solubility of HES in the following order: ChCl/CA is followed by ChCl/OA, ChCl/MA, and ChCl/LA. The results were explained using the molecular structures of DESs, which contain numerous hydrogen bonds. DESs have the most carboxyl groups, which helps them form intermolecular interactions with HES, resulting in the highest solubility. In general, DESs are effective solvents for increasing HES solubility. HES appears to be the HBA in solutions, while CA, OA, MA, and LA appear to be the HBD. The –COOH groups of DES acids interact strongly with HES, whereas LA has a weak interaction, which may result in a stronger interaction of CA with HES, resulting in higher HES solubility in CA-based DES compared to the other investigated DESs. The findings suggest that neoteric green solvents, rather than ILs and organic solvents, are appropriate solvents in pharmaceutical fields. As shown in Fig. 4, the HES solubility in aqueous DES solutions decreases with increasing water content, indicating that the presence of water molecules in the DES disrupted the physical interactions between the constituents of DES, i.e., the solvation or hydration of chloride ion by water molecules, weakening the interaction between acids and ChCl species in solution and reducing the HES solubility. Furthermore, addition of water to the DES solution may improve the polarity, electrical conductivity, and hydrophilicity of the DES + water system because water molecules may easily enter the DES structure and the hydrogen bonds among the DES constituents will be broken, allowing these species to move freely. Polarity, hydrogen bonds, interactions between solvent and solute molecules, enthalpy of fusion, melting point, and other factors can all affect drug solubility (conditions and cohesive energy density).

In addition, the levels of solubility observed for HES in the studied DESs could be due to solute–solvent interactions. Interactions such as H-bonds, van der Waals forces, ion–dipole and dipole–dipole between solute and solvent can be responsible for the solubilization of hydrophobic drugs in a solvent^41,42^. At the atomic level, the drug and DESs can interact with each other mainly via H-bonds interactions. The HES drug has ability to act as HBDs or HBAs, forming H-bonds with DESs. The H-bond is formed between the hydroxyl groups of HES and the hydroxyl or carboxyl and Cl groups of DESs. The solvating power of DESs is remarkable rather than water, because, there are H-bonds and dipole–dipole interactions in water + drug systems. But in DESs + drug systems, there are strong ion–dipole interactions in addition to H-bonds and dipole–dipole interactions. These interactions caused significant increase in the solubility of drugs in DESs systems. On the other hand, it should also be noted that the ability of any DES as a powerful solubilizing agent for a drug is different. The DESs weak intermolecular interactions between the components of the DESs causing strong interactions of DESs-drugs. In this regard, H-bonds interactions between HBA and HBD in DESs were increased with the increase of H-bonds group (hydroxyl and carboxyl groups) and H-bonds interaction of ChCl with second component is weakened^41,43^.

### Thermodynamic models and analysis

Knowing the solubility in pharmacy science enables researchers in this field to recommend appropriate solvents for this job, which helps with the creation of pharmaceuticals as well as improving their qualities. Finding a proper solvent can benefit from modeling solubility data for this reason. The solubility of a solid in a liquid solvent is determined using thermodynamic solid–liquid equilibrium equations.

### Hansen solubility parameters results

In this study, the parameters *δ*_d_, *δ*_p_ and *δ*_h_ were obtained from sources and some were calculated using the Krollen and Hafitzer method for HES drug and DESs which are collected in the Table 4. Differences between drug solubility parameter and DESs are calculated from Eq. (4) and are reported in the Table 5. As can be seen from the results in Table 5, $\Delta \delta$ values indicating a strong interaction between HES and DES (ChCl/CA)) relative to others systems. In other words, the following order reflects the strength of the interaction between the HES and the solvents: HES + ChCl/CA > HES + ChCl/OA > HES + ChCl/MA > HES + ChCl/LA. Finally, the HSP calculation backs up these findings, which are also consistent with the experiment results.

**Table 4:** The calculated HSP for the materials used.

systems	*δ*_d_	*δ*_p_	*δ*_h_	*δ*t
HES	19.6	10.3	13.9	26.1
ChCl/CA	19.7	6.0	17.9	27.3
ChCl/OA	16.3	5.7	14.4	22.5
ChCl/MA	16.3	5.3	13.9	22.1
ChCl/LA	16.1	4.6	16.6	23.6

**Table 5:** The calculated ∆δ for HES drug and solvents (water and DESs).

Systems solute	Water	ChCl/CA	ChCl/OA	ChCl/MA	ChCl/LA
HES	30.1	5.9	8.1	8.3	9.5

## Conclusions

Hesperidin solubility was measured experimentally at *T* = 298.15–313.15 K in the presence of four choline chloride-based DESs. Temperature and DESs weight fractions were all positively correlated with experimental solubility data in aqueous DESs solutions. At the specified temperature, these findings revealed that the order of DESs in increasing HES solubility is as follows: ChCl/CA is followed by ChCl/OA, ChCl/MA, and ChCl/LA. The most important factor in increasing solubility in neat solvents can be hydrogen bonding in the solvent. The drug's experimental solubility data were also correlated using the Van't Hoff–Jouyban–Acree and Modified Apelblat–Jouyban–Acree models. The used models are well compatible with the experimental solubility data based on the percent *ARD* values. The Hansen solubility parameters, on the other hand, were calculated for the investigated systems. In comparison to other systems, the experimental and Hansen solubility parameter results show a strong interaction between HES and DES (ChCl/CA). The thermodynamic analysis of the studied system is also important in the pharmaceutical industry.

## Data Availability

All data generated or analyzed during this study are included in this published article.
